# The lymphocyte-to-monocyte ratio predicts intracranial atherosclerotic stenosis plaque instability

**DOI:** 10.3389/fimmu.2022.915126

**Published:** 2022-07-22

**Authors:** Xiao-Bing Wu, Li-Xin Huang, Zhong-Run Huang, Li-Ming Lu, Bin Luo, Wang-Qing Cai, An-Min Liu, Sheng-Wen Wang

**Affiliations:** ^1^ Department of Neurosurgery, Sun Yat-sen Memorial Hospital, Sun Yat-sen University, Guangzhou, China; ^2^ Department of Neurosurgery, The Eighth Affiliated Hospital, Sun Yat-sen University, Shenzhen, China; ^3^ Clinical Research and Data Center, South China Research Center for Acupuncture and Moxibustion, Medical College of Acu-Moxi and Rehabilitation, Guangzhou University of Chinese Medicine, Guangzhou, China

**Keywords:** intracranial atherosclerotic stenosis, high-resolution vessel wall magnetic resonance imaging, plaque enhancement, lymphocyte-to-monocyte ratio, plaque instability

## Abstract

**Background and purpose:**

Gadolinium enhancement on high-resolution vessel wall imaging (HR-VWI) is an imaging marker of intracranial atherosclerotic stenosis (ICAS) plaque instability. This study aimed to evaluate the relationships between hematological inflammatory indicators and the enhancement of ICAS plaques and to search for hematological indicators that can predict ICAS plaque instability.

**Methods:**

Consecutive adult patients diagnosed with ICAS from April 2018 to December 2021 were recruited retrospectively, and every patient underwent HR-VWI. Plaque enhancement was measured qualitatively and quantitatively. The plaque-to-pituitary stalk contrast ratio (CR) indicated the degree of plaque enhancement. Clinical and laboratory data, including the lymphocyte-to-monocyte ratio (LMR), neutrophil-to-lymphocyte ratio (NLR), and systemic immune inflammation index (SII), were recorded. The hematological inflammatory indicators were compared between ICAS patients with and without plaque enhancement and between patients with and without symptomatic plaque. The hematological inflammatory indicators and the CR were compared using linear regression. Furthermore, receiver operating characteristic curve analysis was performed to assess the discriminative abilities of the inflammatory indicators to predict plaque instability.

**Results:**

Fifty-nine patients were included. The NLR, SII and LMR were significantly correlated with plaque enhancement. The LMR was independently associated with plaque enhancement, and a linear negative correlation was observed between the LMR and CR (R = 0.716, *P* < 0.001). The NLR, LMR, plaque enhancement and CR were significantly associated with symptomatic ICAS, and the LMR and plaque enhancement were independent risk factors for symptomatic ICAS. The optimal cutoff value of the admission LMR to distinguish symptomatic plaque from asymptomatic plaque was 4.0 (80.0% sensitivity and 70.6% specificity).

**Conclusion:**

The LMR was independently associated with ICAS plaque enhancement and showed a linear negative correlation with CR. The LMR and plaque enhancement were independent risk factors for symptomatic ICAS. An LMR ≤ 4.0 may predict ICAS plaque instability.

## Introduction

Intracranial atherosclerotic stenosis (ICAS) is a common cause of ischemic stroke worldwide, accounting for 46.6% of all strokes in China (1–3). Inflammation promotes plaque development and progression, and T lymphocytes, macrophages and interleukins in atherosclerotic plaques play important roles in stroke pathogenesis (4–6). Identifying unstable plaques and treating them in a timely manner is of great significance to patients. High-resolution vessel wall magnetic resonance imaging (HR-VWI) allows the detection of ICAS plaques *in vivo*, and gadolinium plaque enhancement detected by HR-VWI is associated with vulnerable plaques (7, 8). Pathological studies have shown that the macrophage area and microvessel density are independently associated with the gadolinium enhancement of atherosclerotic plaques, indicating enhanced plaque on HR-VWI as local inflammation (9). However, the associations of circulating inflammatory markers with plaque enhancement remains unclear.

The neutrophil-to-lymphocyte ratio (NLR), lymphocyte-to-monocyte ratio (LMR), and systemic immune inflammation index (SII), known as novel circulating inflammatory biomarkers, have been reported to be effective predictors in patients with carotid artery stenosis and coronary atherosclerosis (10–12). The NLR, LMR and SII can predict poor outcomes and prognosis in patients with intracerebral hemorrhage, spontaneous intracerebral hemorrhage, and ischemic stroke (13–18). Moreover, these novel composite inflammatory ratios are more effective and have superior predictive capacities than traditional inflammatory factors (19). Nevertheless, hematological biomarkers that can effectively predict plaque instability are still lacking and worth exploring. We had conducted research before and found that LMR was associated with plaque enhancement of MCA stenosis. This study aimed to evaluate the associations of ICAS plaque enhancement and hematological inflammatory indicators and to search for hematological inflammatory indicators that can predict the instability of ICAS plaques.

## Materials and methods

### Study population and data collection

**Figure 1:**
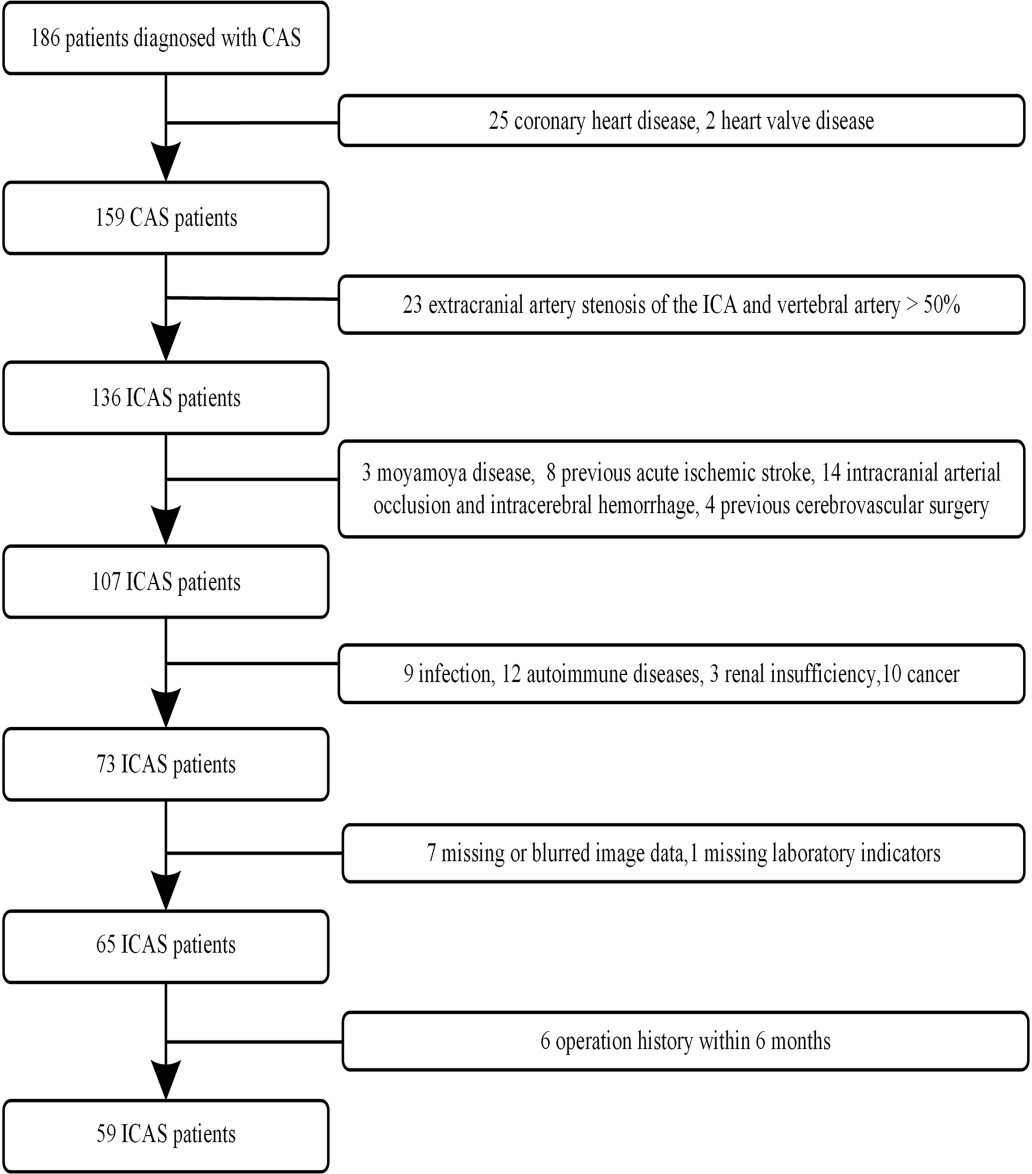
Flow diagram of the selected population.

One hundred eighty-six patients with ICAS were continuously screened from our hospital database between August 2018 and December 2021. All patients with ICAS identified by MR angiography (MRA) or digital subtraction angiography (DSA) received HR-VWI. The clinical data, including age, sex, hypertension, diabetes, current smoking, and drinking status, were recorded. The selection of the study population is presented as a flow diagram (**Figure 1**).

The inclusion criteria were as follows: (1) the patient was more than 18 years old, (2) the images had no artifacts on HR-VWI, and (3) the patient had digital subtraction angiography (DSA) or MRA that confirmed ICAS. The exclusion criteria were as follows: (1) extracranial artery stenosis of the internal carotid artery and vertebral artery > 50%; (2) nonatherosclerotic vasculopathies such as intracranial arteritis, dissections, and moyamoya disease; (3) coronary heart disease and other heart diseases; (4) previous acute ischemic stroke, intracranial arterial occlusion and intracerebral hemorrhage; (5) acute or chronic infection; (6) cancer; (7) history of autoimmune disease; (8) blood disease or serious systemic disease; (9) kidney dysfunction; (10) surgical history within 6 months; and (11) missing image data and laboratory indicators.

### Laboratory measurements

Venous blood samples from the included patients were obtained within 24 hours of admission for complete blood cell count and biochemical analyses. Blood cell analysis was performed by using an automated analytical platform (Sysmex XN9000, Kobe, Japan), which provided the counts of total white blood cells, neutrophils, lymphocytes, monocytes, and platelets. The NLR was calculated as neutrophil counts/lymphocyte counts, the LMR was calculated as lymphocyte counts/monocyte counts, and the SII was calculated as platelets*neutrophils/lymphocytes. Moreover, biochemical analysis was performed using Vitros 5,1 FS (Ortho Clinical Diagnostics), which provided the levels of cholesterol, triglyceride, high-density lipoprotein (HDL), low-density lipoprotein (LDL), apolipoprotein E, and high-sensitivity C-reactive protein (hsCRP).

### Imaging protocol

#### High-resolution vessel wall MR imaging

MR imaging was performed using a 3.0T MR scanner (Achieva TX, Philips Healthcare, Best, the Netherlands) with a 32-channel head coil. Every patient underwent 3D TOF-MRA, DWI, and HR-VWI T1WI (2D/3D) scans. The parameters of the HR-VWI were as follows. On imaging studies performed earlier in the study period, patients underwent 2D T1 HR-VWI (double inversion recovery, DIR) with the following parameters: TR/TE 1000 ms/9 ms; 150 mm× 150 mm field of view; 5 axial/sagittal/coronal slices; resolution 0.45 mm× 0.55 mm in-plane; and 3-mm slice thickness with a scan time of 3 minutes and 45 seconds. Later, in the study, 3D T1 VISTA (motion sensitized driven equilibrium, MSDE) became available in our hospital and was performed with the following parameters: TR/TE 700 ms/35 ms; 200 mm × 251 mm field of view; 171 slices; matrix 252 × 314; 80° flip angle; and resolution 0.8 mm isotropic with an acquisition time of 4 minutes and 55 seconds.

#### Digital subtraction angiography

DSA was performed on the Philips Allura Xper FD20 machine with a nonionic contrast agent (Visipaque, GE Healthcare, Ireland). The acquired data were transferred to the syngo X workspace for 3D reconstruction, and the best working angle was selected for the measurement of the stenosis site and degree. The degree of stenosis was based on the following formula: [1- (D_stenosis_/D_normal_)] × 100% (20).

### Image analysis

ICAS plaques were defined by wall thickening on both unenhanced and contrast-enhanced HR-VWI images (21). Plaque enhancement was evaluated according to the pre- and postcontrast HR-VWI T1-weighted sequences. Plaque enhancement was qualitatively and quantitatively measured on precontrast and postcontrast T1-weighted images by two experienced raters who were blinded to the clinical data. T2-weighted sequences were collectively used to distinguish artifacts such as cerebrospinal fluid and veins. The raters had to determine whether plaque enhancement was present or absent and then measure the mean signal intensity (SI) of the plaque and pituitary stalk (SI_stalk_) on the postcontrast T1-VISTA sequence individually with the Picture Archiving Communication System (PACS). Plaque enhancement was defined as the enhancement of the plaque 5 minutes after the intravenous injection of the gadolinium developing agent and after the signal was highlighted by NMR-enhanced MRI. Disagreements regarding qualitative enhancement were resolved by a third reader. κ values > 0.80 were regarded as excellent for the identification of enhancement (22). The quantitative plaque enhancement was calculated as follows according to previous studies (8, 23): CR = SI_plaque_/SI_stalk_

(**Supplementary Figure I**). The averaged CR measured by two raters was recorded and used for analysis.

Plaque burden was measured on the maximal stenosis site as follows according to previous studies: Plaque burden = (1−lumen area/vessel area)×100% (24, 25).

DWI and fluid-attenuated inversion recovery (FLAIR) sequences were used to determine infarct locations. Lesions were classified as symptomatic and asymptomatic. A plaque was considered symptomatic if it was the only lesion within the ipsilateral territory of the ischemic stroke or it was the most stenotic plaque when multiple plaques were present within the same territory of the ischemic event, transient ischemic attack was not regarded as symptomatic. A plaque was defined as asymptomatic if it was not within the vascular distribution of stoke (26, 27).

### Statistical analysis

Continuous variables, including age and BMI, are presented as the mean ± standard deviation, and the NLR, LMR, and SII are presented as medians (min-max). These parameters were compared using Student’s t test or the Mann–Whitney U test. Categorical variables, sex, hypertension, diabetes, current smoking, drinking status and plaque enhancement are expressed as the number of cases and percentages and were compared using Fisher’s exact or chi-square test.

Univariate analyses of clinical, laboratory, and imaging data of ICAS patients with and without plaque enhancement and then between symptomatic ICAS and asymptomatic ICAS were performed to identify factors associated with plaque enhancement and symptomatic plaques. Factors independently associated with ICAS plaque enhancement and symptomatic plaques were determined by multivariate logistic regression analyses after adjusting for variables with *P* < 0.05 in the univariate comparisons, forward multivariate logistic regression was used to calculated the 95% confidence interval (CI) and odds ratio (OR). Correlation analysis was performed to understand the associations between the CR and inflammatory markers. Furthermore, receiver operating characteristic (ROC) analysis was performed to determine the best cutoff value of the LMR to differentiate symptomatic plaques from asymptomatic plaques. SPSS 23.0 software (SPSS Inc, Chicago, Illinois) was used for the statistical analysis of the data. Two-tailed *P* values < 0.05 were considered statistically significant.

## Results

### Characteristics of the patients

A total of 59 adult patients were recruited in the present study; the mean age was 58.4 ± 9.7 years, and 32 patients (54.2%) were male. Six patients underwent 2D scanning, while 53 underwent 3D scanning. All patients received DWI inspection; 34 lesions were symptomatic, and 25 lesions were asymptomatic. The baseline characteristics are presented in **Table 1**.

**Table 1:** Characteristics of ICAS atherosclerotic plaques with and without enhancement.

	Total (n=59)	Enhancement (n=41)	Nonenhancement (n=18)	P value
Age	58.4 ± 9.7	59.5 ± 9.2	55.7 ± 10.5	0.159
Sex (male)	32 (54.2%)	24 (58.5%)	8 (44.4%)	0.317
BMI	24.7 ± 3.3	24.9 ± 3.1	24.4 ± 3.6	0.603
Hypertension	42 (71.2%)	30 (73.2%)	12 (66.7%)	0.612
Diabetes	21 (35.6%)	12 (29.3%)	9 (50.0%)	0.126
Smoking	19 (32.2%)	14 (34.1%)	5 (27.8%)	0.630
Drinking	10 (16.9%)	7 (17.1%)	3 (16.7%)	1.000
Degree of stenosis				0.043
70%-99%	34 (57.6%)	28 (68.3%)	6 (33.3%)	
50-69%	15 (25.4%)	8 (19.5%)	7 (38.9%)	
< 50%	10 (16.9%)	5 (12.2%)	5 (27.8%)	
Stenosis site				0.735
Anterior circulation	46 (78.0%)	31 (75.6%)	15 (83.3%)	
Posterior circulation	13 (22.0%)	10 (24.4%)	3 (16.7%)	
White blood cell (×10^9^/L)	7.5 ± 1.9	7.8 ± 2.0	6.8 ± 1.6	0.067
Lymphocyte (×10^9^/L)	2.1 ± 0.7	2.0 ± 0.6	2.3 ± 0.7	0.078
Neutrophil (×10^9^/L)	4.6 ± 1.6	5.0 ± 1.7	4.0 ± 1.3	0.004
Monocyte (×10^9^/L)	0.6 ± 0.2	0.6 ± 0.2	0.5 ± 0.2	0.205
Platelet (×10^9^/L)	252.2 ± 53.3	255.7 ± 49.1	244.2 ± 62.7	0.448
Cholesterol (mmol/L)	4.9 ± 1.5	4.9 ± 1.4	5.1 ± 1.7	0.594
Triglyceride (mmol/L)	1.5 (0.6-4.5)	1.6 (0.7-3.7)	1.4 (0.6-4.5)	0.799
HDL (mmol/L)	1.1 ± 0.3	1.1 ± 0.3	1.1 ± 0.2	0.908
LDL (mmol/L)	3.2 ± 1.2	3.1 ± 1.1	3.3 ± 1.3	0.605
Apolipoprotein E (mg/L)	36.9 ± 10.3	36.6 ± 10.6	37.4 ± 10.0	0.802
hsCRP (mmol/L)	1.7 (0.2-64.6)	1.7 (0.2-64.6)	1.9 (0.3-26.9)	0.587
NLR	2.0 (1.0-6.8)	2.5 (1.1-6.8)	1.6 (1.0-4.6)	0.001
LMR	4.2 ± 1.8	3.8 ± 1.6	5.2 ± 1.8	0.005
SII	618.9 ± 376.1	701.8 ± 420.4	506.1 ± 275.3	0.002
Plaque burden	0.81 ± 0.08	0.82 ± 0.08	0.78 ± 0.09	0.034

ICAS, intracranial atherosclerotic stenosis; BMI, body mass index; HDL, high-density lipoprotein; LDL, low-density lipoprotein; hsCRP, high-sensitivity C-reactive protein; NLR, neutrophil-to-lymphocyte ratio; LMR, lymphocyte-to-monocyte ratio; SII, systemic immune-inflammation index.

### Negative linear correlation between LMR and CR

Forty-one patients had stenosis lesions with plaque enhancement on HR-VWI, while 18 patients showed no enhancement. The interreader agreement regarding the presence of plaque enhancement was excellent (κ = 0.803). The median CR was 0.55 in 53 patients, and the median CR in patients with symptomatic ICAS was higher than that in patients with asymptomatic ICAS (0.61 vs. 0.50, *P* = 0.008) (**Figure 3A**).

**Figure 2:**
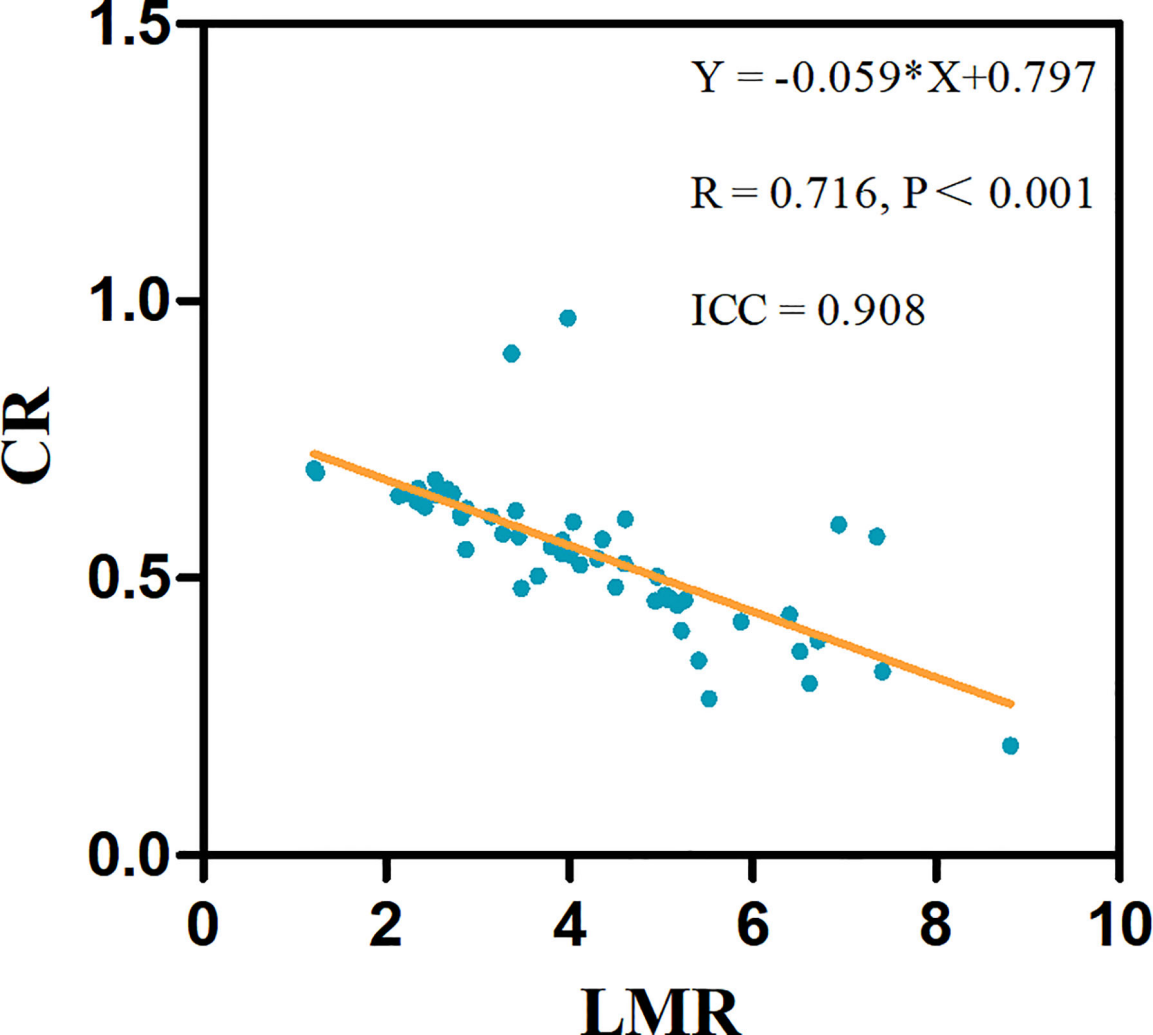
The linear associations significantly differed between the LMR and CR in 53 test patients. The linear regression equations and correlation coefficient R values are provided.

To assess whether the association between the LMR and CR was linear, we constructed linear regression models, revealing a linear negative correlation between the LMR and CR (R = 0.716, p < 0.001) (**Figure 2**).

### A low LMR as an independent predictor of ICAS plaque enhancement

The clinical data of the patients and stenosis sites did not differ between patients with and without plaque enhancement. The levels of neutrophils, NLR, SII and plaque burden (*P* = 0.004, *P* = 0.001, *P* = 0.002, *P* = 0.034, respectively) were higher, while the LMR (*P =* 0.005) was lower in patients with plaque enhancement than those without plaque enhancement (**Table 1**). The levels of total white blood cells, lymphocyte, monocyte, hsCRP, cholesterol, triglycerides, HDL, LDL, and apolipoprotein E did not differ between patients with and without plaque enhancement. Forward multiple logistic regression showed that the LMR (OR: 0.617, 95% CI, 0.428-0.889, *P* = 0.010) was independently associated with plaque enhancement (**Table 2**).

**Table 2:** Forward multiple logistic regression analysis of ICAS plaque enhancement.

Variable	Odds Ratio	95% Confidence Interval	P Value
LMR	0.617	0.428-0.889	0.010

LMR, lymphocyte-to-monocyte ratio.

### A low LMR as an independent predictor of symptomatic ICAS

**Figure 3:**
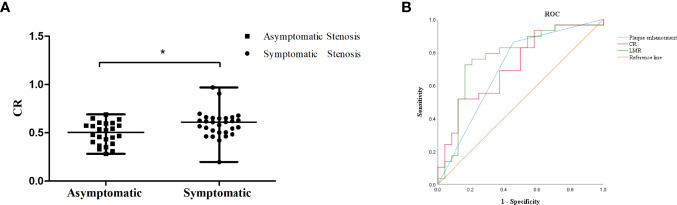
**(A)** The CR of symptomatic ICAS was higher than that of asymptomatic ICAS, * p < 0.05.**(B)** ROC curve for determining the presence of symptomatic stenosis. The AUC of LMR was 0.765 (95% CI: 0.636-0.894, *P* = 0.001). The sensitivity was 80.0%, and the specificity was 70.6%. The AUC of plaque enhancement was 0.686 (95% CI: 0.544-0.829, *P* = 0.015). The sensitivity was 85.3%, and the specificity was 52.0%. The AUC of CR was 0.714 (95% CI: 0.574-0.854, *P* = 0.008). The sensitivity was 51.7%, and the specificity was 87.5%.

The Neutrophil, monocyte, NLR, LMR, plaque enhancement, and CR were associated with symptomatic ICAS (**Table 3** and **Figure 3A**). The multivariate logistic regression analysis revealed that the LMR (OR: 0.625, 95% CI, 0.421-0.928, *P* = 0.020) and plaque enhancement (OR: 4.074, 95% CI, 1.078-15.392, *P* = 0.038) were independently associated with symptomatic ICAS (**Table 4**).

**Table 3:** Characteristics of ICAS plaques with and without symptoms.

	Total (n=59)	Symptomatic Stenosis (n=34)	Asymptomatic Stenosis (n=25)	*P* Value
Age	58.4 ± 9.7	59.0 ± 9.8	57.5 ± 9.6	0.574
Sex (male)	32 (54.2%)	21 (61.8%)	11 (44.0%)	0.176
BMI	24.7 ± 3.3	24.2 ± 3.0	25.4 ± 3.4	0.190
Hypertension	42 (71.2%)	26 (76.5%)	16 (64.0%)	0.296
Diabetes	21 (35.6%)	14 (41.2%)	7 (28.0%)	0.296
Smoking	19 (32.2%)	10 (29.4%)	9 (36.0%)	0.593
Drinking	10 (16.9%)	6 (17.6%)	4 (16.0%)	1.000
Degree of stenosis				0.045
70-99%	34 (57.6%)	24 (70.6%)	10 (40.0%)	
50-69%	15 (25.4%)	5 (14.7%)	10 (40.0%)	
< 50%	10 (16.9%)	5 (14.7%)	5 (20.0%)	
Stenosis site				0.747
Anterior circulation	46 (78.0%)	26 (76.5%)	20 (80.0%)	
Posterior circulation	13 (22.0%)	8 (23.5%)	5 (20.0%)	
White blood cell (×10^9^/L)	7.5 ± 1.9	7.9 ± 2.1	7.0 ± 1.6	0.072
Lymphocyte (×10^9^/L)	2.1 ± 0.7	2.0 ± 0.6	2.2 ± 0.7	0.241
Neutrophil (×10^9^/L)	4.1 (2.6-8.4)	5.0 (2.6-9.0)	3.5 (2.7-7.3)	0.018
Monocyte (×10^9^/L)	0.6 ± 0.2	0.6 ± 0.2	0.5 ± 0.2	0.026
Platelet (×10^9^/L)	250.2 ± 53.3	254.6 ± 51.8	249.0 ± 56.3	0.696
Cholesterol (mmol/L)	4.9 ± 1.5	4.8 ± 1.5	5.1 ± 1.5	0.414
Triglyceride (mmol/L)	1.5 (0.6-4.5)	1.5 (0.6-3.9)	1.5 (0.8-4.5)	0.443
HDL (mmol/L)	1.1 ± 0.3	1.1 ± 0.3	1.2 ± 0.3	0.166
LDL (mmol/L)	3.2 ± 1.2	3.1 ± 1.2	3.3 ± 1.2	0.623
Apolipoprotein E (mg/L)	36.9 ± 10.3	35.9 ± 10.7	38.2 ± 9.9	0.394
hsCRP (mmol/L)	1.7 (0.2-64.6)	1.4 (0.2-64.6)	1.9 (0.3-26.9)	0.624
NLR	2.0 (1.0-6.8)	2.6 (1.2-6.8)	1.7 (1.0-4.6)	0.011
LMR	4.2 ± 1.8	3.5 ± 1.4	5.1 ± 1.8	0.001
SII	491.1 (94.3-2333.5)	568.3 (246.5-2333.5)	410.8 (94.3-1114.4)	0.053
Plaque burden	0.81 ± 0.08	0.82 ± 0.08	0.79 ± 0.09	0.119
Plaque enhancement	41 (69.5%)	29 (85.3%)	12 (48.0%)	0.002

ICAS, intracranial atherosclerotic stenosis; BMI, body mass index; HDL, high-density lipoprotein; LDL, low-density lipoprotein; hsCRP, high-sensitivity C-reactive protein; NLR, neutrophil-to-lymphocyte ratio; LMR, lymphocyte-to-monocyte ratio; SII, systemic immune-inflammation index.

**Table 4:** Forward multiple logistic regression analysis of symptomatic stenosis.

Variable	Odds Ratio	95% Confidence Interval	*P* Value
LMR	0.625	0.421-0.928	0.020
Plaque enhancement	4.074	1.078-15.392	0.038

LMR, lymphocyte-to-monocyte ratio.

The LMR was obviously lower in the patients with symptomatic ICAS than in those with asymptomatic ICAS. The cutoff value of the LMR to differentiate symptomatic ICAS from asymptomatic ICAS on the ROC curve was 4.0, with a 80.0% sensitivity and 70.6% specificity, *P* = 0.001, and the area under the curve (AUC) was 0.765 (**Figure 3B**).

## Discussion

Our study showed that neutrophils, the NLR, the LMR, the SII and the plaque burden were significantly correlated with ICAS plaque enhancement. The LMR was independently associated with ICAS plaque enhancement and showed a linear negative correlation with the CR. Low LMR was an independent risk factor for symptomatic ICAS. The optimal cutoff value of LMR to distinguish symptomatic ICAS was 4.0.

Atherosclerotic plaque enhancement on HR-VWI indicates local inflammation (9, 28). Whether plaque enhancement is associated with circulating inflammatory indicators remains unclear. This study showed that neutrophils, the NLR, the LMR and the SII were significantly correlated with ICAS plaque enhancement. A previous study showed that the levels of peripheral blood neutrophils were associated with the presence of ICAS (29). The NLR, LMR and SII are sensitive inflammatory markers in peripheral blood that can predict poor outcomes and prognosis for patients with intracerebral hemorrhage, spontaneous intracerebral hemorrhage, and ischemic stroke (13–18). Furthermore, this study indicated that the LMR was independently associated with plaque enhancement and showed a linear negative correlation with the CR, which reflecting the degree of plaque enhancement. The CR is an effective indicator of the degree of plaque enhancement and has a significant correlation with plaque instability (8). Additionally, this study also showed that the mean LMR was 3.8 in ICAS patients with plaque enhancement. A large sample study of 5000 healthy adults in China showed that the mean LMR was 5.0 (30). The mean LMR in the ICAS plaque enhancement group was significantly lower than that in healthy adults. Our results suggested that ICAS plaque enhancement lead to a systemic inflammatory process, and a higher degree of plaque enhancement is correlated with more serious peripheral inflammation.

Several studies have shown that plaque enhancement is a valuable imaging marker of plaque instability (7, 8, 31). A previous study showed that higher plaque burden identified on HR-VWI is independently associated with recurrent ischemic stroke (25). This study showed that higher plaque burden was associated with plaque enhancement, also revealed that higher plaque burden may showed more plaque instability.

This study confirmed that the plaque enhancement and the enhancement degree of plaque was obviously more extensive in patients with symptomatic ICAS than in those with asymptomatic ICAS. We referred previous studies that a plaque was considered symptomatic only when the lesion of DWI was positive, and the previous transient ischemic attack without DWI-positive lesion was not regarded as symptomatic (26, 27).

No hematological indicators showed an ability to predict ICAS plaque instability. This study found that the NLR and SII of symptomatic ICAS were significantly higher and that the LMR was significantly lower than those of asymptomatic ICAS. The LMR was independently associated with symptomatic ICAS, and the ROC curve analysis showed that the cutoff value of the LMR to differentiate symptomatic ICAS from asymptomatic ICAS was 4.0, with a sensitivity of 80.0% and a specificity of 70.6%. The sensitivity and specificity of the LMR to differentiate symptomatic ICAS from a symptomatic was high, which indicates that an LMR ≤ 4.0 suggests a high likelihood of plaque instability. A previous study showed that an LMR ≤ 4.8 could be a predictor of coronary artery disease (CAD) and that the LMR could be used as a marker of the coronary plaque burden in CAD (12). The levels of peripheral blood inflammatory indicators were higher in patients with symptomatic ICAS than in those with asymptomatic ICAS, which may support the idea that inflammation promotes ICAS plaque formation and progression to ischemic events.

Dyslipidemic conditions were not related to plaque enhancement or symptomatic ICAS in our study. A previous study suggested that the presence of ICAS enhancement was significantly associated with dyslipidemic conditions (32). Our results were inconsistent with the previous findings, perhaps due to the incomplete exclusion criteria and to the fact that some patients in this study had been on lipid-lowering therapy for a period of time. In addition, previous studies have shown that higher degrees of stenosis are correlated with more pronounced plaque enhancement, indicating that inflammation promotes plaque progression (33). This study also showed that the stenosis in patients with plaque enhancement was more extensive than that in patients without enhancement.

Several limitations should be considered in this study. First, this was a single-center retrospective study with a relatively small sample size, all cases were from patients admitted to our hospital, which lead to bias for patient selection and statistical analysis. Second, infection at admission may have been somewhat inadequately assessed despite our exclusion criteria due to the retrospective design. Third, although two independent raters were in excellent agreement regarding plaque enhancement, the simultaneous 2D and 3D T1 sequences in different patients and limited HR-VWI spatial resolution may have affected their assessment of plaque enhancement. This study did not evaluate plaque morphology, which may be associated with ischemic stroke due to limited spatial resolution. Fourth, the inflammatory mechanism may have been related to plaque enhancement, and the reason why the LMR reduction was independently associated with plaque enhancement is not clear. In addition, we found that not all inflammatory indicators were associated with plaque enhancement, perhaps due to the different sensitivities of each index to ICAS plaque enhancement and the relatively small sample size of this study. Finally, although the LMR and plaque enhancement were independently associated with symptomatic ICAS, whether they can predict plaque instability requires further studies to dynamically assess.

## Conclusion

ICAS plaque enhancement may lead to a systemic manifestation of inflammation, and the LMR is independently associated with plaque enhancement and shows a linear negative correlation with the CR. An LMR ≤ 4.0 may predict plaque instability, which needs further research for confirmation.

## Data Availability Statement

The original contributions presented in the study are included in the article/**Supplementary Material**. Further inquiries can be directed to the corresponding authors.

## Ethics Statement

The studies involving human participants were reviewed and approved by Sun Yat-sen Memorial Hospital. Written informed consent for participation was not required for this study in accordance with the national legislation and the institutional requirements.

## Author Contributions

A-ML and S-WW contributed to the study design. X-BW and L-XH wrote the main manuscript text. L-XH and Z-RH prepared figures. All authors participated in the interpretation and collection of the data. All authors reviewed the manuscript.

## Funding

This study was supported by the National Natural Science Foundation of China (Grant No. 81901339).

## Conflict of Interest

The authors declare that the research was conducted in the absence of any commercial or financial relationships that could be construed as a potential conflict of interest.

## Publisher’s Note

All claims expressed in this article are solely those of the authors and do not necessarily represent those of their affiliated organizations, or those of the publisher, the editors and the reviewers. Any product that may be evaluated in this article, or claim that may be made by its manufacturer, is not guaranteed or endorsed by the publisher.

## Supplementary material

Supplementary Figure A 64-year-old woman was diagnosed with moderate basilar artery stenosis. **(A)** MRA showed eccentric stenosis of the basilar artery; **(B)** DSA showed stenosis of the basilar artery; **(C)** No infarction was found on DWI; **(D**, **E)** Mean signal value of basilar artery plaque and pituitary stalk on postcontrast T1-weighted.

## Abbreviation

HR-VWI, high-resolution vessel wall imaging; ICAS, intracranial atherosclerotic stenosis; CR, The plaque-to-pituitary stalk contrast ratio; LMR, lymphocyte-to-monocyte ratio; NLR, neutrophil-to-lymphocyte ratio; SII, systemic immune inflammation index; MRA, MR angiography; DSA, digital subtraction angiography; HDL, high-density lipoprotein; LDL, low-density lipoprotein; hsCRP, high-sensitivity C-reactive protein; DIR, double inversion recovery; MSDE, motion sensitized driven equilibrium; 3D TOF-MRA, three-dimensional time-of-flight MR angiography; DWI, diffusion-weighted imaging; HR-VWI T1WI, HR-VWI T1-weighted sequences; SI, the mean signal intensity; SI_stalk_, the mean signal intensity of pituitary stalk; PACS, the Picture Archiving Communication System; 3D T1-VISTA, three-dimensional T1 volumetric isotropic turbo spin echo acquisition; FLAIR, fluid-attenuated inversion recovery; BMI, Body Mass Index; ROC, receiver operating characteristic; AUC, the area under the curve; CAD, coronary artery disease; NMR-enhanced MRI, nuclear magnetic resonance-enhanced Magnetic Resonance Imaging.
