# Supplementary figures and images for: The lymphocyte-to-monocyte ratio predicts intracranial atherosclerotic stenosis plaque instability
Source: Front Immunol. 2022 Jul 22;13:915126. doi: 10.3389/fimmu.2022.915126 (PMC9355723; doi:10.3389/fimmu.2022.915126)

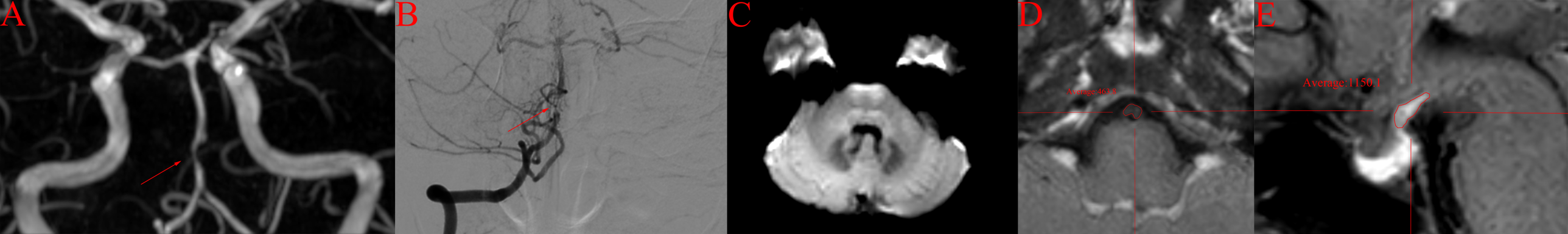

Supplement: Supplementary Figure — A 64-year-old woman was diagnosed with moderate basilar artery stenosis. (A) MRA showed eccentric stenosis of the basilar artery; (B) DSA showed stenosis of the basilar artery; (C) No infarction was found on DWI; (D, E) Mean signal value of basilar artery plaque and pituitary stalk on postcontrast T1-weighted. [file Image_1.jpeg]
